# Supplementary material for: Hippocampal sequences represent working memory and implicit timing
Source: Cell Rep. Author manuscript; Available in PMC 2025 Nov 5. (PMC12588557; doi:10.1016/j.celrep.2025.116383)
Supplement: 1 [file NIHMS2120112-supplement-1.pdf]

Cell Reports, Volume *44*

Supplemental information

Hippocampal sequences represent working memory and implicit timing

Conor C. Dorian, Jiannis Taxidis, Dean V. Buonomano, and Peyman Golshani

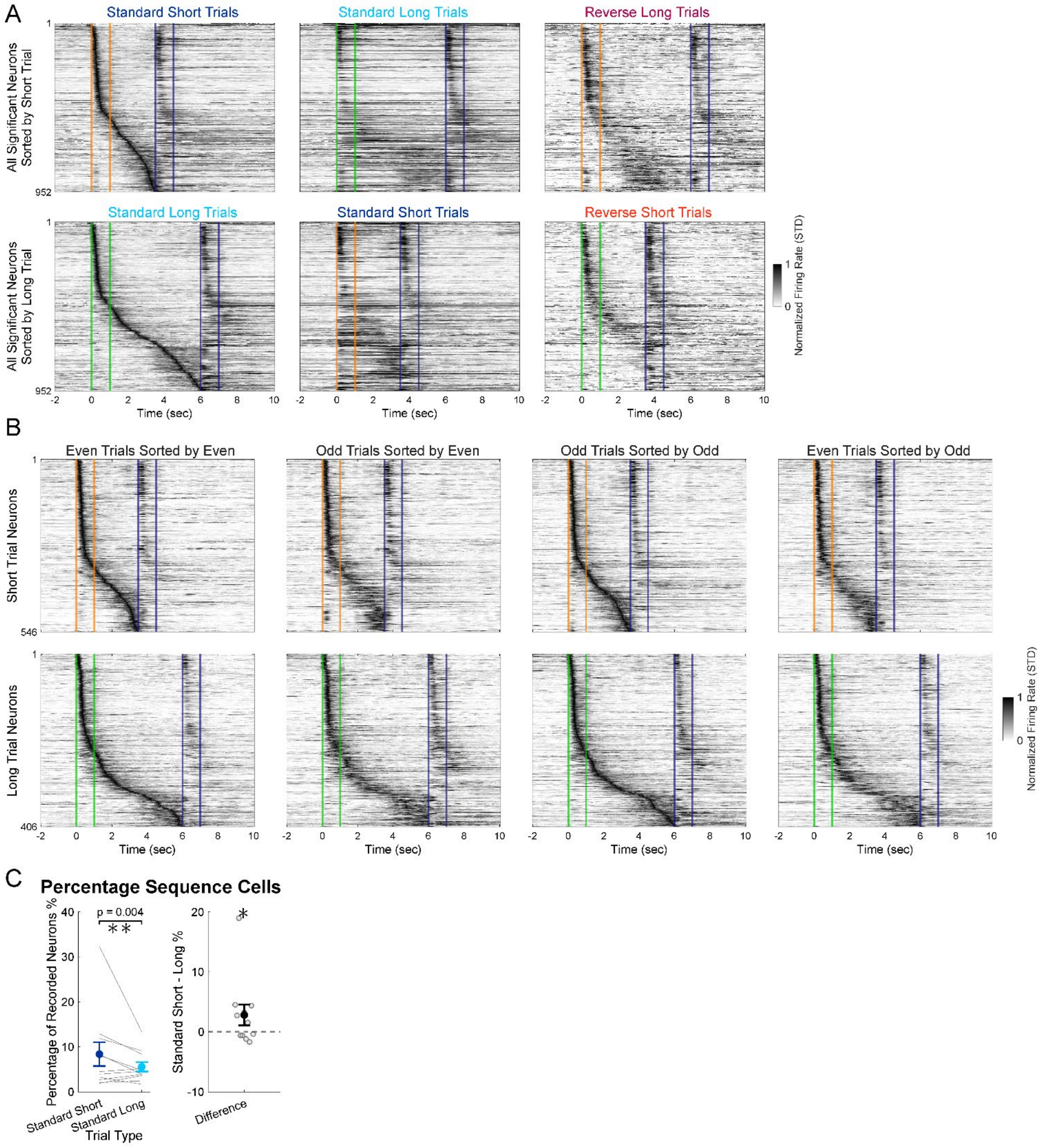

Supplemental Figure 1: Sequential activity in CA1 persists when pooling both neuron populations and in even/odd trial validation. A) Same as Figure 2D, but all significant neurons have been pooled together and resorted for both panels on the left. As in Figure 2D, the middle and right panels are sorted the same as left panels. **B)** Even/odd trial validation of the original sequences in Figure 2D. For even trials sorted by even, average traces were resorted and visualized only using even trials. For odd trials sorted by even, the average traces of odd trials were visualized using the same sorting as the panels to the left. The 4 panels on the right are sequences when sorted by the odd trials. **C)** Percentage of recorded neurons that passed criteria to be sequence cells. Lines and dots represent animal averages. Two-Way ANOVA animal and day for the 44 recording sessions, p = 0.0041.

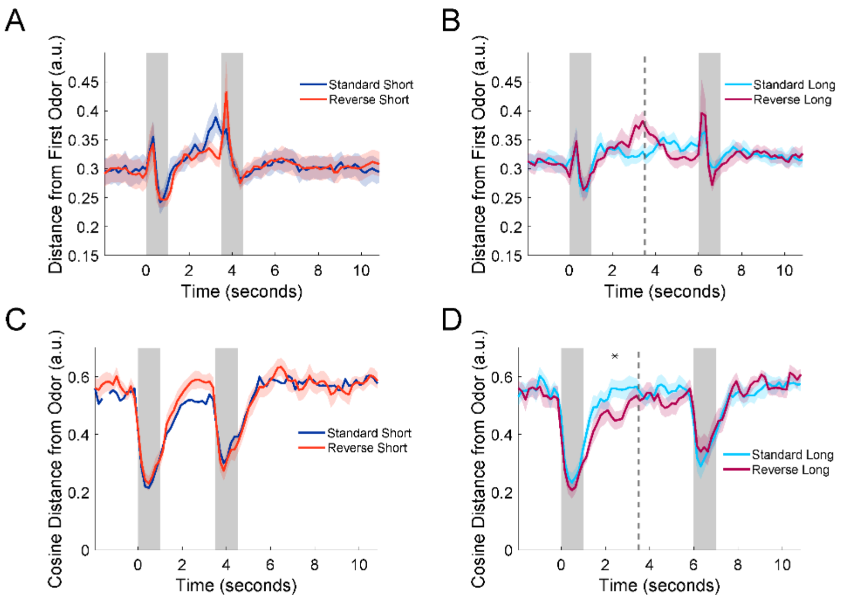

Supplemental Figure 2. Neural trajectory distance from first odor and cosine distances. A-B) Same as Figure 3 B-C, but calculating distance from the average neural activity during the entire one second first odor period. **C-D)** Same as A and B but using cosine distance instead of euclidian distance. Asterisks represent bins of 1/6 second that were significantly different (Two-Way ANOVA over animal and day, corrected for multiple comparisons with Benjamini-Hochberg procedure, $p < 0.01$).

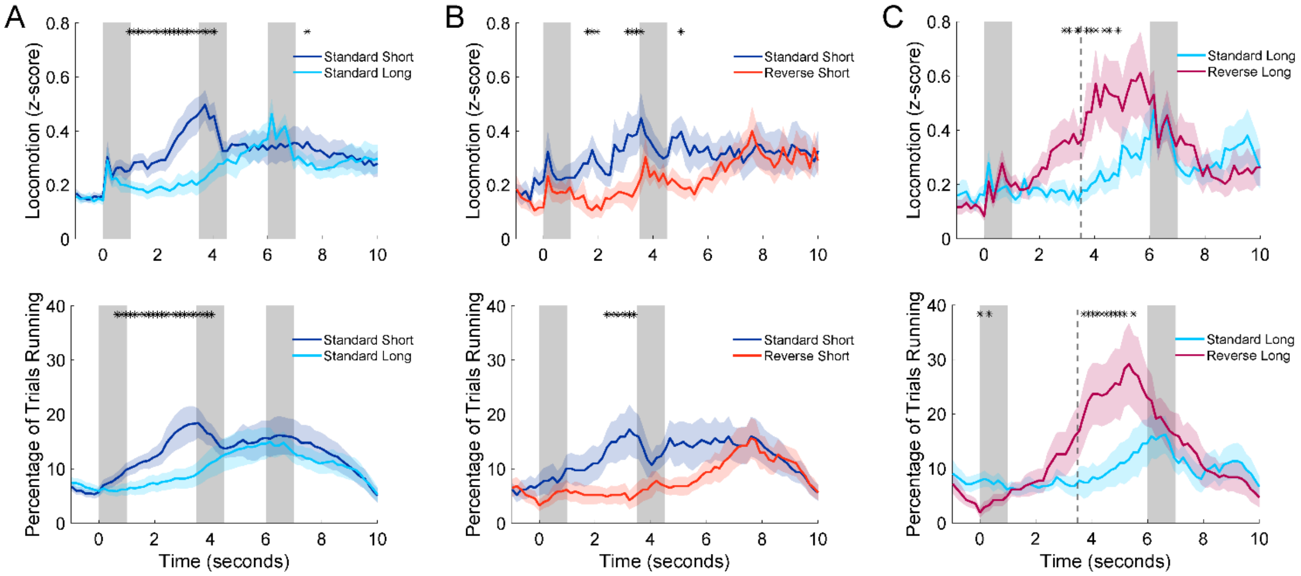

Supplemental Figure 3: Running differences during delays showed evidence of implicit timing. A) Top panel: locomotion is z-scored voltage signal from motion sensor binned to 1/6 second. Bottom panel: percentage of trials with running in a given bin. For both panels, comparison was made between all standard short and standard long trials from the last 2 standard days and first 2 reverse days. The gray bar at time point 0 is the 1st odor for both trial types, while the one at 3.5 is the 2nd odor for standard-short trials and the one at 6 is the 2nd odor for standard-long trials. Thick lines represent the mean of 44 recording sessions (11 mice across 4 days), and shaded area represents standard error of the mean. Asterisks represent bins of 1/6 second that were significantly different (Two-Way ANOVA over animal and day, corrected for multiple comparisons with Benjamini-Hochberg procedure, $p < 0.01$). **B)** Same as (A), but comparison between standard-short and reverse-short trials from first 2 reverse days with the number of trials balanced by taking the nearest neighbor standard trial for each reverse trial. Statistics were the same as (A), but for only 22 recording sessions. **C)** Same as (B), but comparison between standard-long and reverse-long trials. The dotted line represents the expected 2nd odor arrival on reverse-long trials.

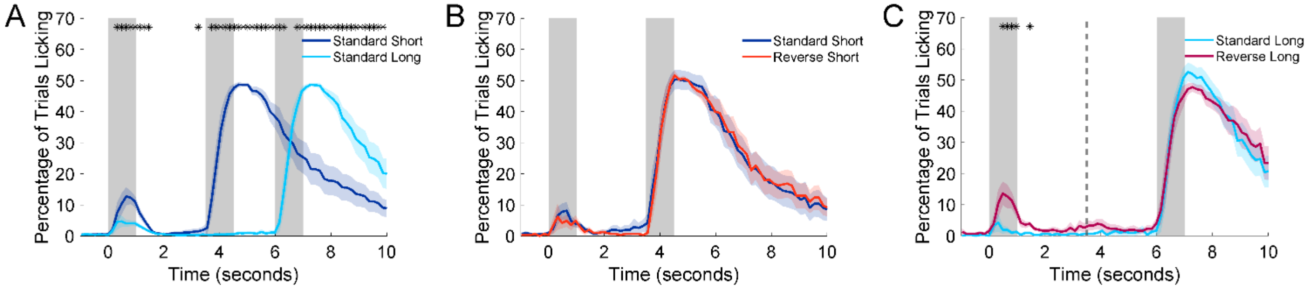

Supplemental Figure 4: Licking patterns do not show evidence of implicit timing. A-C) Same as Supplemental Figure 1 D-F but comparing the percentage of trials when the mouse was licking. All analyses were identical to running. Thick lines represent the mean of 44 recording sessions (11 mice across 4 days), and shaded area represents standard error of the mean. Asterisks represent bins of 1/6 second that were significantly different (Two-Way ANOVA over animal and day, corrected for multiple comparisons with Benjamini-Hochberg procedure, $p < 0.01$).

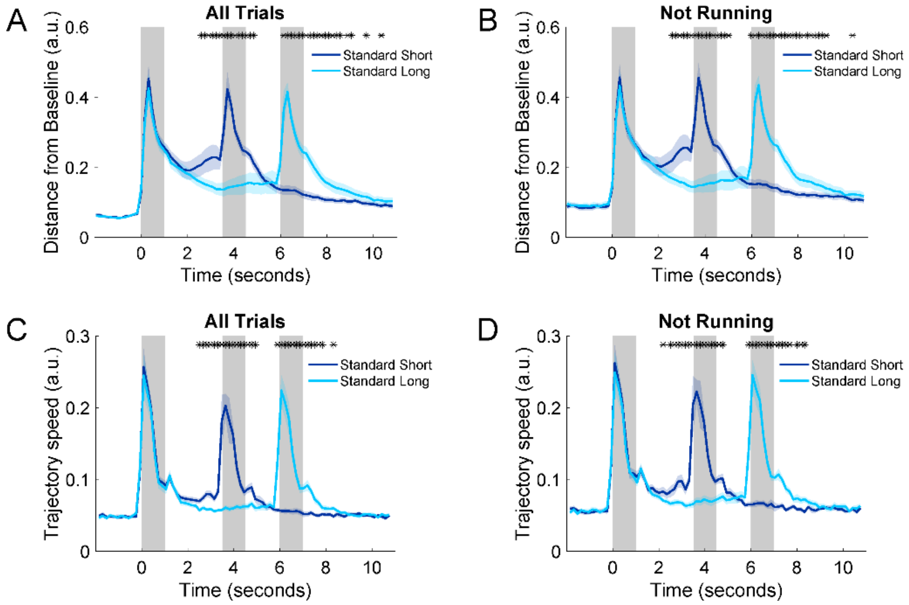

Supplemental Figure 5: Locomotion running patterns did not explain effects of neural trajectories. A) Same as Figure 3 B-C but comparing standard short and standard long trials. **B)** Same as (A) but excluding trials in which a 1-second period of running occurred during the delay period. **C)** Same as Figure 3 D-E but comparing standard short and standard long trials. **D)** Same as (C) but excluding running trials.

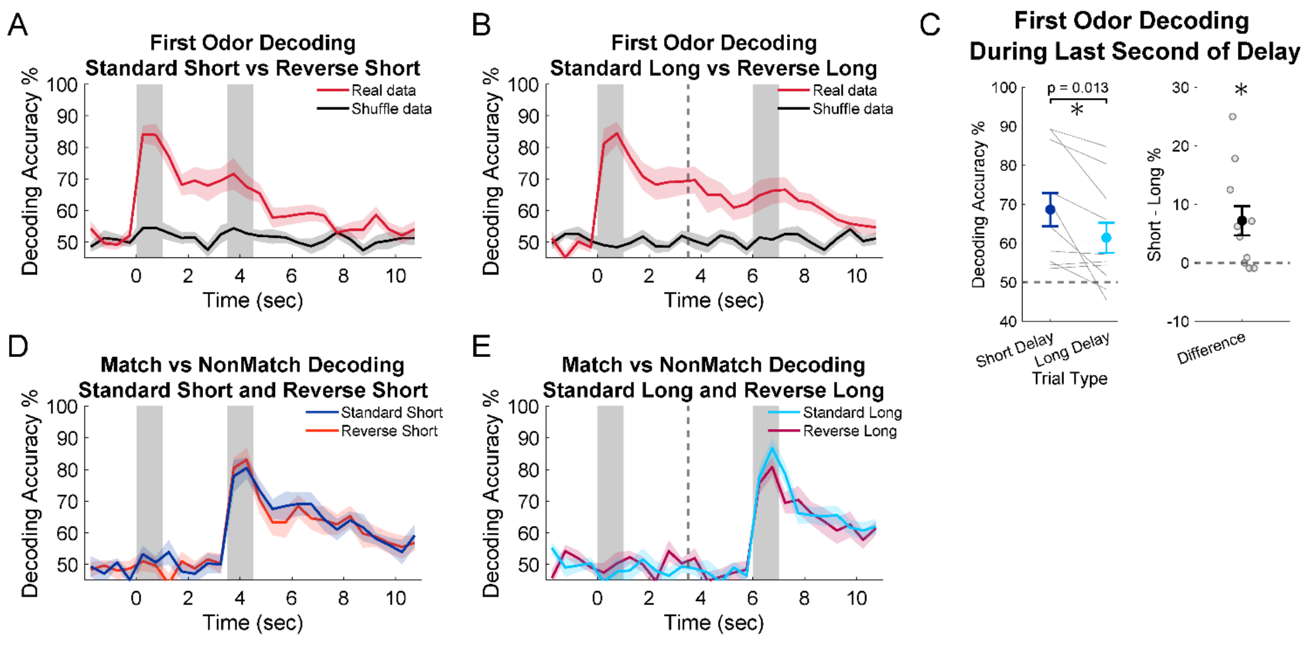

Supplemental Figure 6: Better decoding of first odor identity at end of short vs long delay, while match vs nonmatch decoding comparable. A) Binary support vector machine decoding of first identity between standard short and reverse short trials. Bin size is 0.5 seconds, and the black represents accuracy when trial assignment was randomly shuffled. The number of trials were balanced as done in all plots in Figure 3 and 4. **B)** Same as A but for standard long and reverse long trials. **C)** Quantifications of decoding accuracy from panels A and B during the last second of delay (2.5 to 3.5 seconds from panel A, and 5 to 6 seconds from panel B). Lines and dots represent animal averages. Two-Way ANOVA over animal and day for the 22 recording sessions with reverse trials, p = 0.013. **D-E)** Similar to A and B but decoding whether the trial was a match or non-match. Two-Way ANOVA on each bin revealed no bins with a significant difference.
